# Nomogram for predicting occurrence and prognosis of liver metastasis in elderly colorectal cancer patients: a population-based study

**DOI:** 10.3389/fonc.2023.1295650

**Published:** 2024-01-04

**Authors:** Qi Wang, Kexin Shen, Bingyuan Fei, Mengqiang Wei, Zhongshi Xie

**Affiliations:** Department of Gastrointestinal Colorectal and Anal Surgery, China-Japan Union Hospital of Jilin University, Changchun, China

**Keywords:** colorectal cancer, liver metastasis, nomogram, SEER database, elderly patients

## Abstract

**Introduction:**

This study aimed to explore independent risk and prognostic factors in elderly patients with colorectal cancer liver metastasis (ECRLM) and generate nomograms for predicting the occurrence and overall survival (OS) rates of such patients.

**Method:**

Elderly colorectal cancer patients (ECRC) from 2010 to 2015 in the Surveillance, Epidemiology, and End Results (SEER) database were included in this study. External validation relied on Chinese patients from the China-Japan Union Hospital of Jilin University. Univariate and multivariate logistic regression analyses were employed to identify liver metastasis (LM) risk variables, which were used to create a nomogram to estimate LM probabilities in patients with ECRC. Univariate and multivariable Cox analyses were performed to identify prognostic variables and further derive nomograms that could predict the OS of patients with ERCLM. Differences in lifespan were assessed using the Kaplan–Meier analysis. Finally, the quality of the nomograms was verified using decision curve analysis (DCA), calibration curves, and receiver operating characteristic curves (ROC).

**Result:**

In the SEER cohort, 32,330 patients were selected, of those, 3,012 (9.32%) were diagnosed with LM. A total of 188 ECRLM cases from a Chinese medical center were assigned for external validation. LM occurrence can be affected by 13 factors, including age at diagnosis, marital status, race, bone metastases, lung metastases, CEA level, tumor size, Grade, histology, primary site, T stage, N stage and sex. Furthermore, in ECRLM patients, 10 variables, including age at diagnosis, CEA level, tumor size, lung metastasis, bone metastasis, chemotherapy, surgery, N stage, grade, and race, have been shown to be independent prognostic predictors. The results from both internal and external validation revealed a high level of accuracy in predicting outcomes, as well as significant clinical utility, for the two nomograms.

**Conclusion:**

We created two nomograms to predict the occurrence and prognosis of LM in patients with ECRC, which would contribute significantly to the improvement in disease detection accuracy and the formulation of personalized cures for that particular demographic.

## 1 Introduction

Currently, colorectal cancer (CRC) is the third most prevalent malignant neoplasm and the second leading cause of cancer-related mortality (1). The older population have a higher susceptibility to cancer, making them the primary demographic affected by CRC (2, 3). Advancements in screening and treatment methods have improved the overall survival (OS) rates of young individuals with CRC. However, this positive trend is not observed in older patients (4, 5). Moreover, the OS of CRC patients may be substantially reduced at the onset of metastasis (6, 7). The liver is the most common organ for distant metastasis of CRC (8). At the time of initial diagnosis, approximately 20–30% of CRC patients have liver metastasis (LM), and as the malignancy progresses, approximately half of the patients develop LM. LM has a dramatic and harmful effect on patients with CRC, with a median duration of survival of approximately 6 months (9–11). This issue worsens dramatically when LM occurs in elderly colorectal cancer (ECRC) patients who defined by age surpass 65 years old (12). Despite experiencing a greater burden of CRC and poorer prognosis than other age groups, elderly patients with colorectal cancer liver metastasis (ECRLM) are often underrepresented in clinical trials and receive inadequate treatment in clinical practice.

To enhance the OS rate of this population, it is imperative to expeditiously identify ECRC patients with liver metastasis trends, assess their survival outcomes, and devise individualized therapeutic strategies. CRC treatment and prognosis were based on tumor-node-metastasis (TNM) stage system of American Joint Committee on Cancer (AJCC). Despite having identical TNM staging, numerous patients with colorectal cancer liver metastasis (CRLM) exhibit substantial variations in prognosis (13). Given the complex characteristics of patients with ECRLM, it is imperative to devise a novel model that can effectively predict its incidence and prognosis. Nomograms have been extensively used as a clinical prediction model that combines many variables to determine the probability of a specific clinical occurrence (14). The utilization of nomograms in clinical practice can aid surgeons in recognizing ECRC patients with LM and provide prognostic information regarding the 1-, 3-, and 5-year survival odds for such populations, thereby enabling the development of personalized treatment strategies for this specific cohort. Multiple studies have shown that CRC patients with synchronous LM have a higher prevalence and worse outcomes than those with metachronous LM (15). Therefore, this study focused on exploring synchronous liver metastases.

This study analyzed the risk and prognostic variables associated with ECRLM by selecting an aged patient group with CRC from the SEER database. Subsequently, two nomograms were created to estimate the probability and OS of patients with ECRLM. To demonstrate the beneficial effects of the nomograms, a Chinese population dataset was used for external validation. These two nomograms possess significant reference values for disease diagnosis and treatment.

## 2 Materials and methods

### 2.1 Study population

Using SEER∗Stat 8.3.6 (www.seer.cancer.gov/seerstat), we were able to get information about ECRC patients from the SEER database. Recording of metastatic locations in the SEER database, including the liver, bone, brain, and lungs, was not completed until 2010. Thus, the study population consisted of older patients who were given a pathological diagnosis of CRC between 2010 and 2015 and whose follow-up records were complete. Patients with ECRC were selected using the criteria outlined in the 3rd edition of the International Classification of Diseases for Oncology (ICD‐O‐3) based on the primary location of the tumor (C18.0-C18.7, C19.9, and C20.09). Participants were disqualified from the study if they fulfilled any of the following criteria: (I) crucial details such as tissue type, TNM stage, or demographic information were not available. (II) CRCs were not primary tumors. (III) The metastatic status of CRC was unclear. (IV)The survival time of the patients was either missing or recorded at 0 month. (V) CRC diagnosis was based only on autopsy or postmortem examinations. As an external validation cohort, 188 patients were retrospectively recruited from the China-Japan Union Hospital of Jilin University between July 2013 and December 2019. The last follow-up took place in January 2023. Both the SEER and Chinese populations were subjected to the above-mentioned criteria for admission and exclusion. The absence of personal information from the public data released in the SEER database obviates the need for ethical committee approval and informed consent. This retrospective study of a Chinese cohort was approved by the Ethics Committee of the China-Japan Union Hospital of Jilin University, guaranteeing that the study met the ethical standards of the Helsinki Declaration.

**Figure 1:**
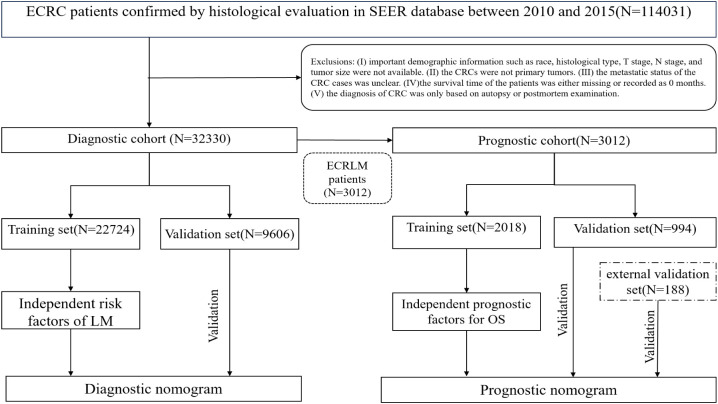
The procedure for selecting patients and the process involved in this research.

In this study, 32,330 patients with ECRC were included in the diagnostic cohort to investigate the risk factors associated with LM. A diagnostic nomogram was generated to predict the odds of LM. Three thousand and twelve patients with ECRC suffered from LM of the 32,330 ECRC patients. These patients were then assigned to the prognosis cohort for analysis of the elements that influence prognosis, and a prognostic nomogram was devised to predict survival rates in patients with ECRLM. **Figure 1** shows the patient selection procedure and the process involved in this research.

### 2.2 Data selection

We selected 15 variables to study the risk of LM development in patients with ECRC, including age at diagnosis, race, sex, marital status, insurance status, tumor size, histology, grade, T stage, N stage, primary site, bone metastases, lung metastases, brain metastases, and CEA levels. In the analysis of the prognosis of ECRLM, the survival analysis encompassed two additional factors: surgery and chemotherapy. Individuals are placed into distinct racial groups, including black, white, and others, based on their diverse ethnic backgrounds. The patients were divided into three different categories according to the anatomical position of the tumor: left colon, right colon, and rectum. Tumor size is typically determined by its maximal cross-sectional diameter. Early studies split the population into distinct cohorts according to tumor size: those with 5 cm or fewer tumors and those with tumors larger than 5 cm, a classification we also employed (16, 17). Statistics from the SEER database indicated that around 70% of all occurrences occur in people aged 65 and above, with over 40% of patients aged 75 and above (4). Based on this criterion, patients were divided into two groups: those aged 75 and above and those aged 65–75 years. OS was defined as the time between diagnosis and death.

### 2.3 Statistical analyses

Quantitative variables were denoted by mean ± standard deviation (SD), whereas categorical variables were typically described using numbers as well as percentages (N, %). All statistical analyses in the current investigation were performed using SPSS (version 27.0) and R software 4.2.2(https://www.r-project.org/). If the p-value is 0.05 or less (both sides), the result will be statistically significant. To improve the precision of our model, we applied R software to randomly divide the research population into two separate subsets: a training cohort and a validation cohort, with a ratio of 7:3. To examine whether there was a statistically noteworthy distinction within both collections of baseline information, the χ2 test was used. Univariate logistic regression analysis was performed to ascertain pertinent variables associated with LM. Variables that exhibited a significance level (P < 0.05) in the univariate analysis were subsequently put into the multivariate logistic regression analysis, which ultimately determined the independent risk variables for ECRLM patients (P < 0.05). Additionally, we obtained the odds ratio (OR) and 95% confidence interval (CI) to demonstrate the relationship between the risk factors and LM occurrence. Similarly, COX regression analysis was performed on patients with ECRLM as a way to explore potential prognostic variables for this specific patient population. To show the effect of a given prognostic factor on OS, we used the hazard ratio (HR) and the corresponding 95% CIs. Finally, relying on the identified independent risk variables and prognostic factors, the “RMS” program package within the R software will be recruited to generate the relevant nomograms. Using the internal validation cohort, internal validation was executed on the diagnostic and prognostic nomograms, encompassing the ROC curve, calibration curve, and DCA curve. Similarly, external validation was performed on the prognostic nomogram using an external validation cohort. Furthermore, all patients with ECRLM were separated into high-, intermediate-, and low-risk subgroups according to the tertile of their aggregate points provided by the prognostic nomogram. Subsequently, Kaplan–Meier survival analysis and the log-rank test were performed to investigate the disparity in OS among the three groups.

## 3 Results

### 3.1 Characterization of included cases

In our study, 32,330 patients with ECRC were enrolled from the SEER database, and 188 patients with ECRLM were recruited for external validation. **Table 1** outlines the demographic and clinicopathological features of ECRC patients with or without LM, including 15,517 (48.00%) male and 16,813(52.00%) female cases. In terms of ethnicity, White constituted the majority (N = 26,196, 81.03%). The age range at diagnosis was virtually identical, which involved 16,583 patients (51.29%) aged >75 and 15,747 individuals (48.71%) aged 65–75. Regarding tumor size, the vast majority of individuals (N = 20410, 63.13%) had tumors ≤5 cm. In addition, adenocarcinoma (N = 24073, 74.46%) and N0 (N =18602, 57.54%) were frequently found in patients with ECRC. Furthermore, as demonstrated in **Table 1**, the chi-squared test confirmed that the difference in demographic and clinicopathological characteristics of ECRC patients we include was unintentional.

**Table 1:** Demographic and clinicopathological characteristics of ECRC patients in SEER with or without LM.

Characteristics	Number of patients (n, %)				
	Total N=32330	Training cohort N=22724	Inclusion Validation cohort N=9606		*P*-value
Age					
65-75	15747 (48.71)	11036 (48.57)	4711 (49.04)		0.433
>75	16583 (51.29)	11688 (51.43)	4895 (50.96)		
Insurance status					
Insured	32140 (99.41)	22584 (99.38)	9556 (99.48)		0.305
Uninsured	190 (0.59)	140 (0.62)	50 (0.52)		
Marital status					
Married	16920 (52.32)	11885 (52.30)	5035 (52.42)		0.852
Unmarried ^a^	15410 (47.66)	10839 (47.70)	4571 (47.58)		
Gender					
Female	16813 (52.00)	11787 (51.87)	5026 (52.32)		0.458
Male	15517 (48.00)	10937 (48.13)	4580 (47.68)		
Race					
Black	3042 (9.41)	2159 (9.50)	883 (9.19)		0.638
Other ^b^	3092 (9.56)	2162 (9.51)	930 (9.68)		
White	26196 81.03)	18403 (80.98)	7793 (81.13)		
Primary site					
Left	9573 (29.61)	6727 (29.60)	2846 (29.63)		0.429
Rectum	4374 (13.53)	3110 (13.69)	1264 (13.16)		
Right	18383(56.86)	12887 (56.71)	5496 (57.21)		
Grade					
Grade I	2298 (7.11)	1595 (7.02)	703 (7.32)		0.085
Grade II	23048 71.29)	16209 (71.33)	6839 (71.20)		
Grade III	5800 (17.94)	4120 (18.13)	1680 (17.49)		
Grade IV	1184 (3.66)	800 (3.52)	384 (4.00)		
Histology					
Adenocarcinoma	24073(74.46)	16907 (74.40)	7166 (74.60)		0.710
Other	8257 (25.54)	5817 (25.60)	2440 (25.40)		
T stage					
T1	2971 (9.19)	2075 (9.13)	896 (9.33)		0.655
T2	4948 (15.30)	3478 (15.31)	1470 (15.30)		
T3	18624(57.61)	13135 (57.80)	5489 (57.14)		
T4	5787 (17.90)	4036 (17.76)	1751 (18.23)		
N stage					
N0	18602 57.54)	13032 (57.35)	5570 (57.98)		0.126
N1	8751 (27.07)	6223 (27.39)	2528 (26.32)		
N2	4977 (15.39)	3469 (15.27)	1508 (15.70)		
Tumor size					
≤5cm	20410(63.13)	14319 (63.01)	6091 (63.41)		0.500
>5cm	11920(36.87)	8405 (36.99)	3515 (36.59)		
Bone metastasis					
No	32191(99.57)	22622 (99.55)	9569 (99.61)		0.424
Yes	139 (0.43)	102 (0.45)	37 (0.39)		
Brain metastasis					
No	32282(99.85)	22696 (99.88)	9586 (99.79)		0.073
Yes	48 (0.15)	28 (0.12)	20 (0.21)		
Liver metastasis					
No	29318(90.68)	20648 (90.86)	8670 (90.26)		0.086
Yes	3012 (9.32)	2076 (9.14)	936 (9.74)		
Lung metastasis					
No	31467(97.33)	22118 (97.33)	9349 (97.32)		0.965
Yes	863 (2.67)	606 (2.67)	257 (2.68)		
CEA					
Negative	18058 (55.86)	12724 (55.99)	5334 (55.53)		0.441
Positive	14272 (44.14)	10000 (44.01)	4272 (44.47)		

a Includes single, separated, widowed, and divorced.

b Includes American Indian/Alaska Native and Asian or Pacific Islander.

### 3.2 Risk factors that influence LM and the diagnostic nomogram

**Figure 2:**
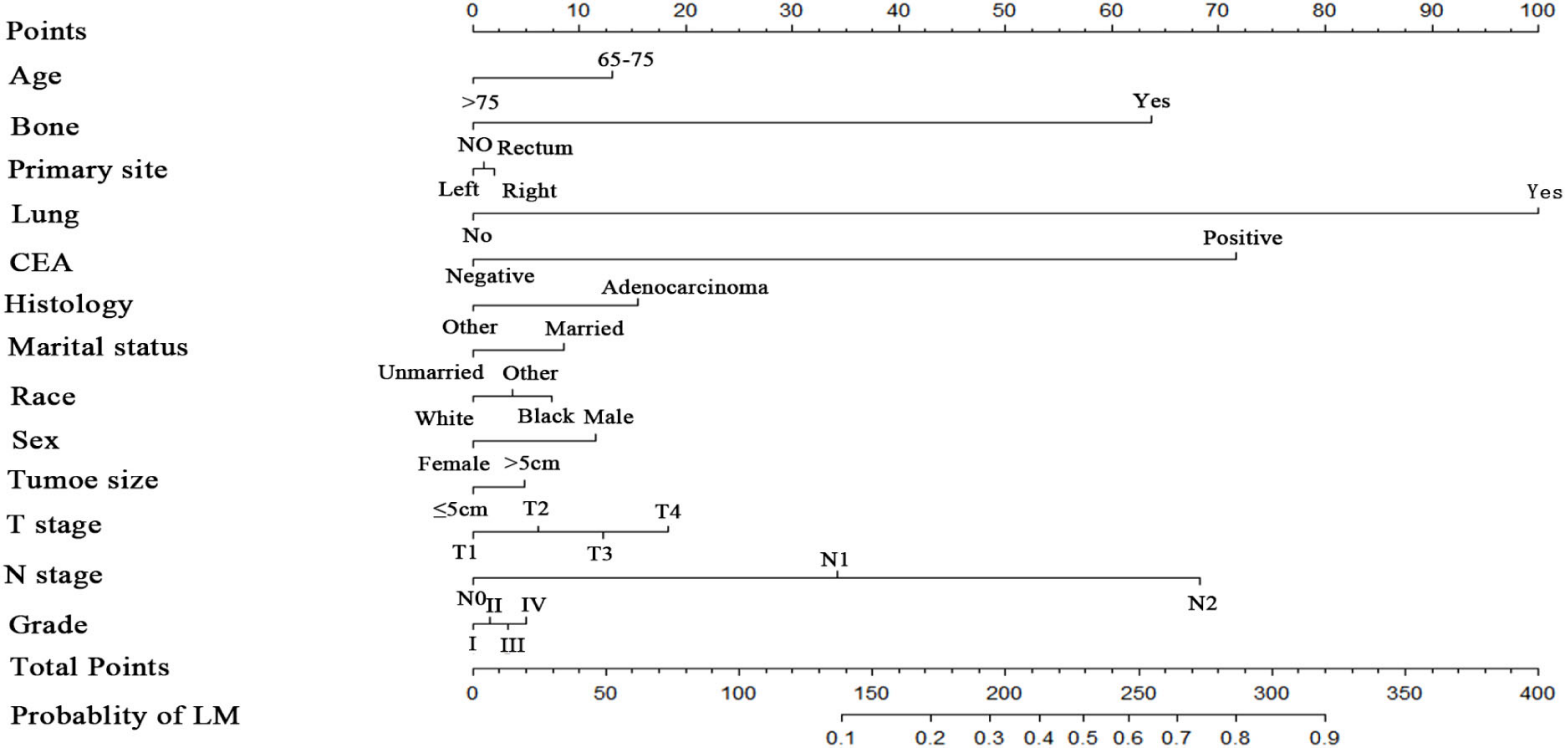
Diagnostic nomogram for figuring out the probability of LM in ECRC patients.

**Figure 3:**
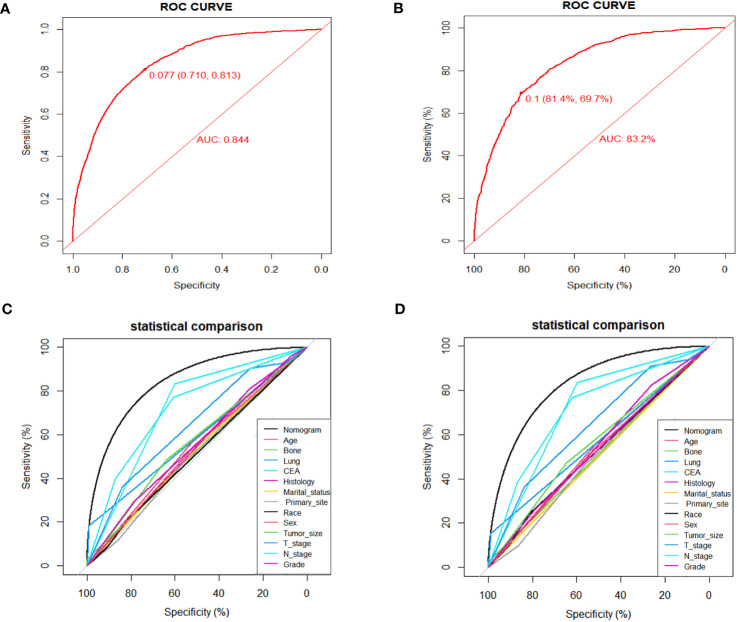
ROC curves for the diagnostic nomogram in the training cohort **(A)** and the validation cohort **(B)**; Comparison of AUC between diagnostic nomogram and all factors in the training cohort **(C)** and validation cohort **(D)**.

**Figure 4:**
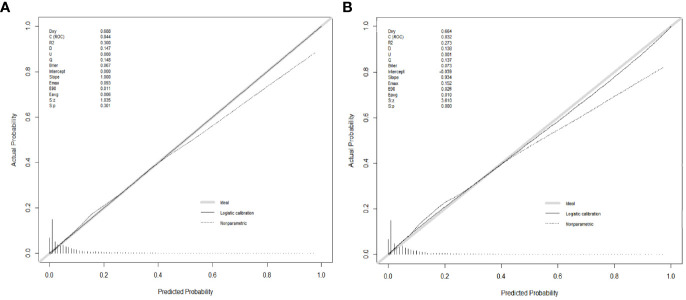
Calibration curves for the diagnostic nomogram in the training cohort **(A)** and the validation cohort **(B)**.

**Figure 5:**
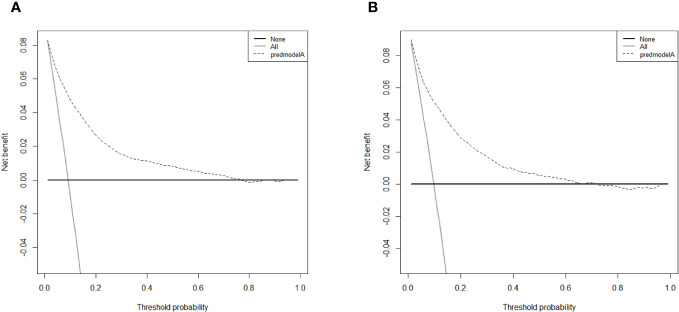
DCA curves for the diagnostic nomogram in the training cohort **(A)** and the validation cohort **(B)**.

Numerous variables were identified as independent risk factors for developing LM in newly diagnosed patients with ECRC through univariate and multivariate logistic regression analyses (**Table 2**). These variables included age at diagnosis, marital status, race, sex, bone metastases, lung metastases, CEA levels, tumor size, histology, primary site, Grade, T stage, and N stage. Subsequently, a diagnostic nomogram was established to determine the risk of LM in patients with ECRC (**Figure 2**). The AUC was 0.844 (95% CI, 0.836–0.852) in the training cohort and 0.832 (95% CI, 0.819–0.845) in the validation cohort (**Figures 3A, B**). These results suggest that the nomogram exhibited good discriminatory performance. It is noteworthy that each risk variable produced its own ROC curve and that the AUC of a single variable was considerably lower than that of the nomogram (P < 0.05), confirming that the whole model had a stronger predictive capacity than a single clinical aspect (**Figures 3C, D**). The calibration curves exhibited a robust alignment between the predictions generated by the nomogram and the real-world results (**Figures 4A, B**). Furthermore, DCA findings indicated that the predictive nomogram exhibited substantial net benefits, indicating its perfect clinical application in predicting LM in patients newly diagnosed with ECRC (**Figures 5A, B**).

**Table 2:** Univariate and multivariate logistic analysis to determine the independent risk factors of ECRLM patients.

Characteristics	Univariate analysis			Multivariate analysis		
	OR	CI	P	OR2	CI2	P2
Age						
65-75	Reference			Reference		
75	0.69	0.63-0.76	<0.001	0.74	0.69-0.82	<0.001
Bone metastasis						
NO	Reference			Reference		
Yes	16.54	11.06-24.72	<0.001	4.71	2.82-7.88	<0.001
Brain metastasis						
No	Reference			Reference		
Yes	5.55	2.56-12.03	<0.001	1.19	0.46-3.07	0.722
Insurance						
Insured	Reference			Reference		
Uninsured	1.11	0.64-1.92	0.72	NA	NA	NA
CEA						
Negative	Reference			Reference		
Positive	7.54	6.69-8.49	<0.001	5.54	4.88-6.30	<0.001
Grade						
I	Reference			Reference		
II	1.76	1.40-2.22	<0.001	1.41	1.09-1.82	0.009
III	2.60	2.04-3.312	<0.001	1.40	1.07-1.84	0.016
IV	2.58	1.895-3.51	<0.001	1.32	0.93-1.87	0.121
Histology						
Adenocarcinoma	Reference			Reference		
Other	0.66	0.59-0.74	<0.001	0.67	0.59-0.76	<0.001
Lung metastasis						
No	Reference			Reference		
Yes	20.56	17.31-24.43	<0.001	10.46	8.60-12.74	<0.001
Marital status						
Married	Reference			Reference		
Unmarried ^a^	0.84	0.77-0.92	<0.001	0.79	0.71-0.88	<0.001
N stage						
N0	Reference			Reference		
N1	3.73	3.31-4.20	<0.001	2.82	2.47-3.22	<0.001
N2	8.18	7.26-9.23	<0.001	5.36	4.65-6.17	<0.001
Primary site						
Left	Reference			Reference		
Rectum	1.43	1.23-1.67	<0.001	1.45	1.22-1.73	<0.001
Right	1.13	0.98-1.31	0.09	1.24	1.04-1.47	0.015
Race						
Black	Reference			Reference		
Other ^b^	1.41	1.22-1.62	<0.001	1.10	0.94-1.29	0.239
White	0.96	0.82-1.12	0.579	0.72	0.6-0.86	<0.001
Sex						
Female	Reference			Reference		
Male	1.26	1.15-1.38	<0.001	1.32	1.18-1.47	<0.001
T stage						
T1	Reference			Reference		
T2	0.22	0.16-0.31	<0.001	0.19	0.13-0.26	<0.001
T3	1.28	1.07-1.53	0.008	0.46	0.37-0.58	<0.001
T4	3.11	2.58-3.76	<0.001	0.69	0.55-0.87	0.002
Tumor size						
≤5cm	Reference			Reference		
>5cm	1.65	1.51-1.81	<0.001	1.13	1.02-1.26	0.021

a Includes single, separated, widowed, and divorced.

b Includes American Indian/Alaska Native and Asian or Pacific Islander.

### 3.3 Prognostic factors and nomogram in patients with ECRLM

**Figure 6:**
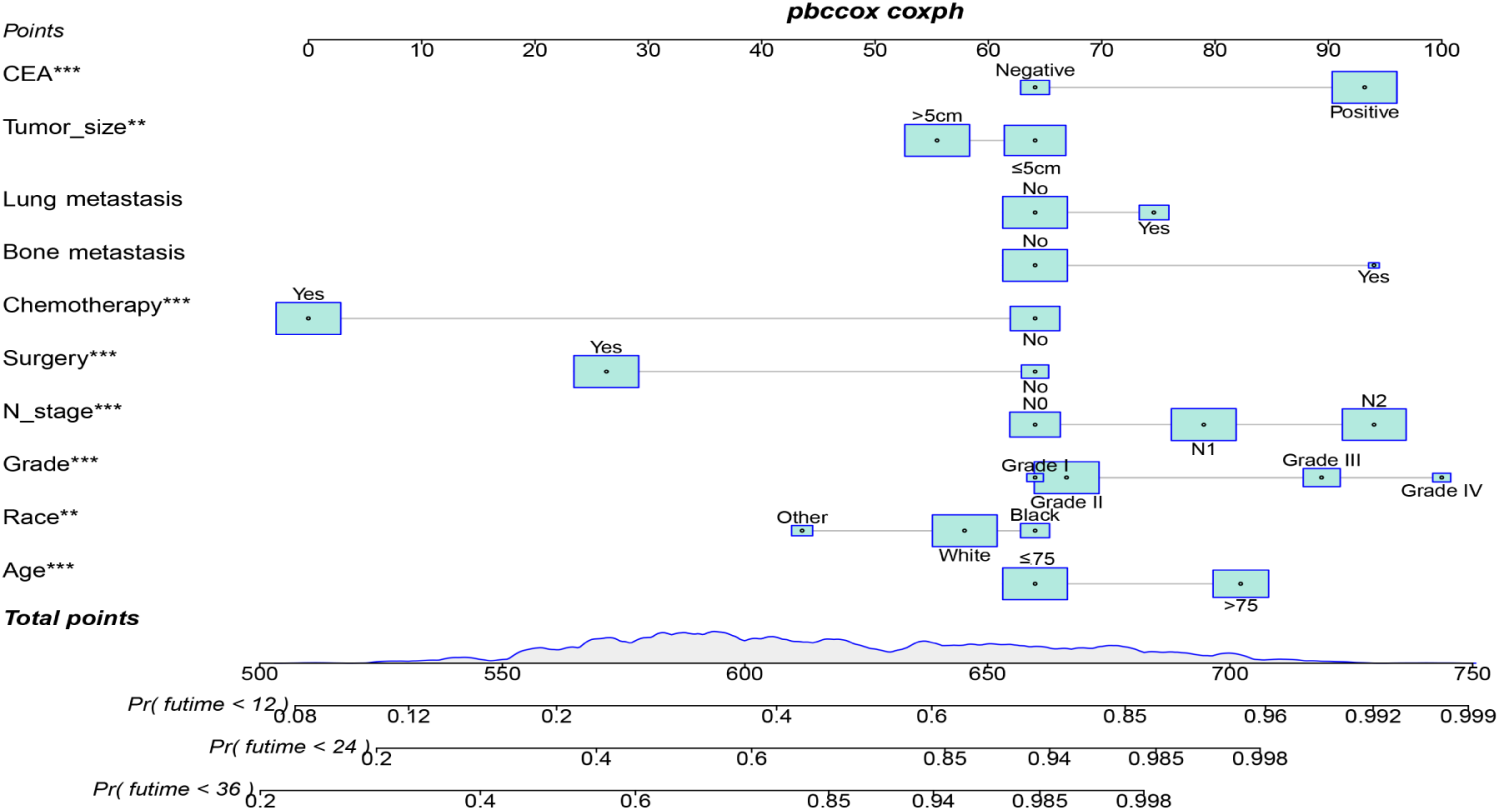
Prognostic nomogram for predicting 12-, 24- and 36-month OS in ECRC patients.

**Figure 7:**
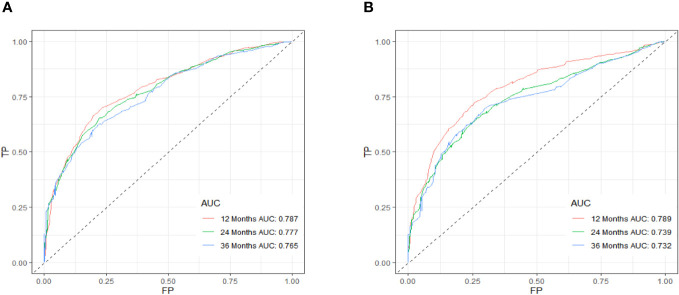
ROC curve of the prognostic nomogram for 12, 24, and 36 months in the training cohort **(A)** and the validation cohort **(B)**.

**Figure 8:**
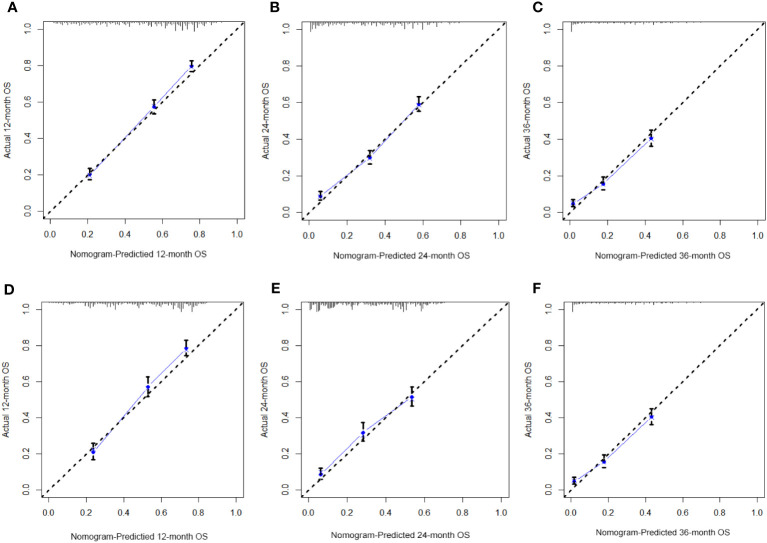
The calibration curves of the prognostic nomogram for the 1-year, 2-year, and 3-year in the training cohort **(A–C)** and in the validation cohort **(D–F)**.

**Figure 9:**
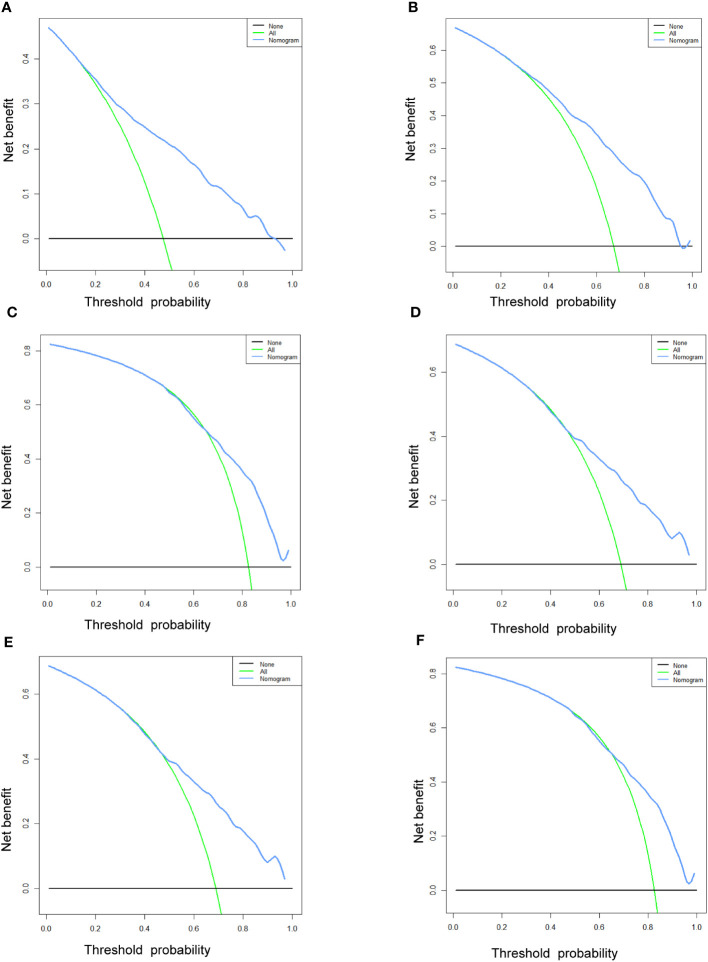
The DCA curves of the prognostic nomogram for the 1-year, 2-year, and 3-year in the training cohort **(A–C)** and in the validation cohort **(D–F)**.

**Figure 10:**
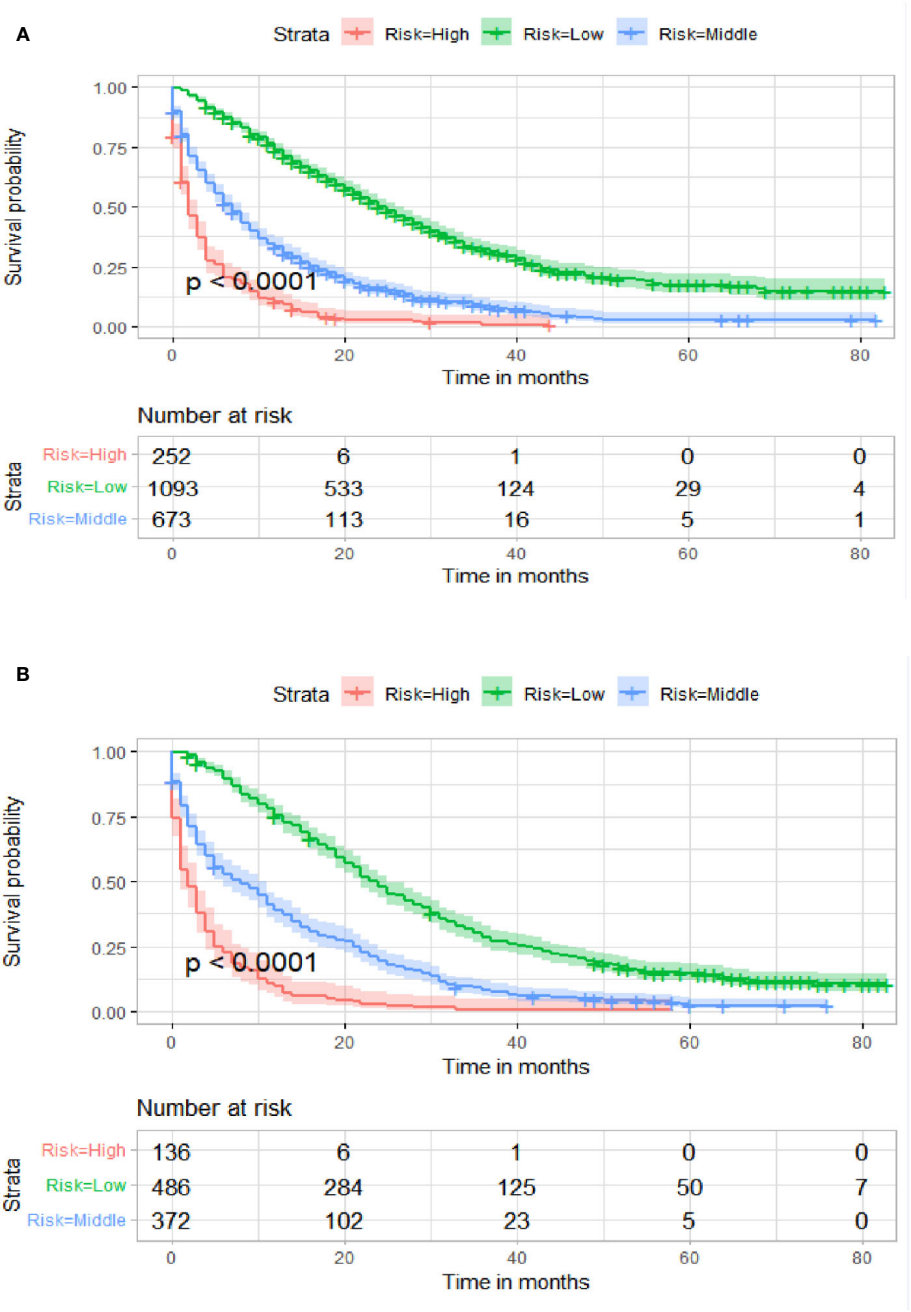
Kaplan–Meier survival curves of three subgroups in the training cohort **(A)** and validation cohort **(B)**.

**Figure 11:**
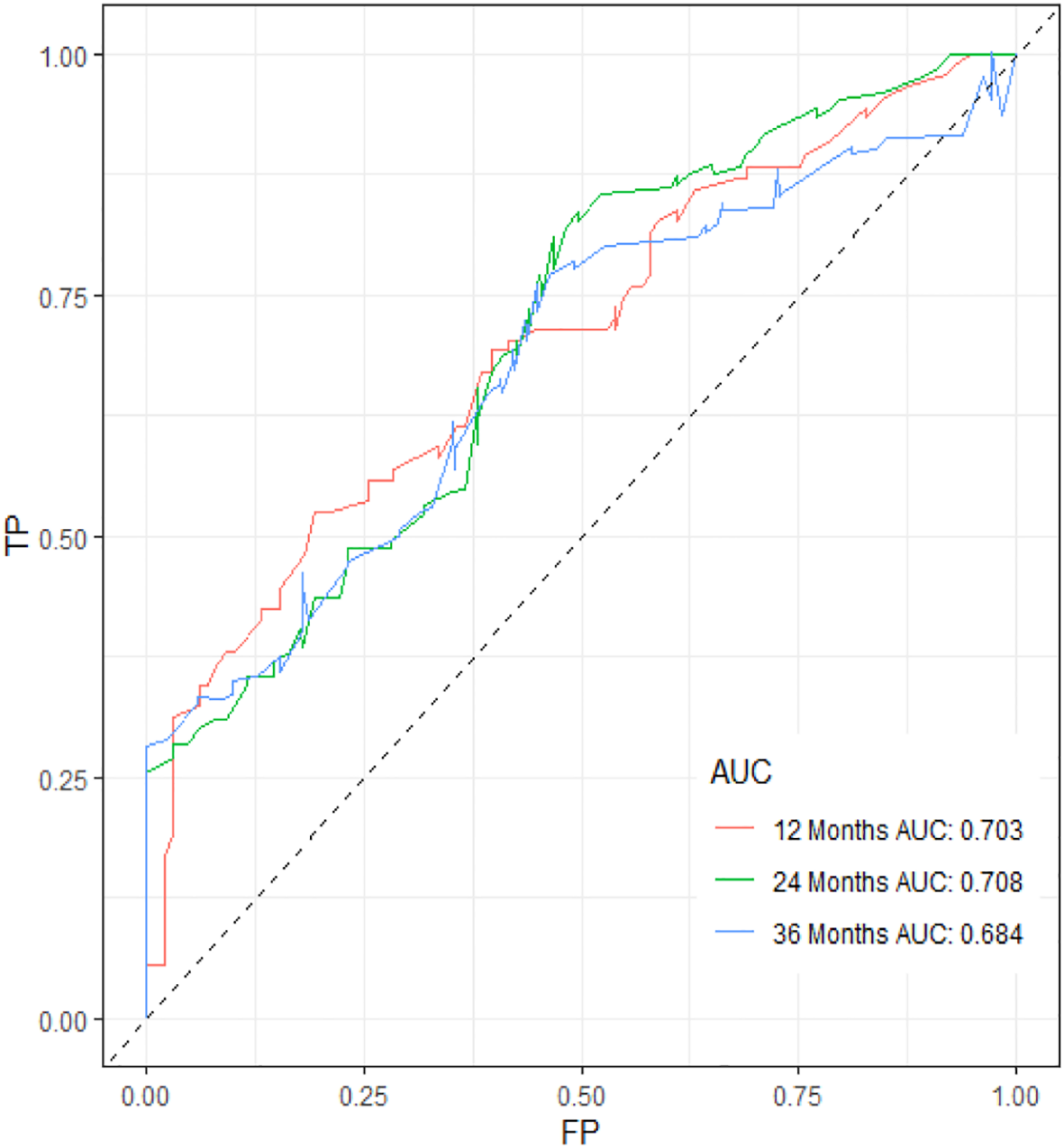
ROC curve of the prognostic nomogram for 12, 24, and 36 months in the external validation set.

**Figure 12:**
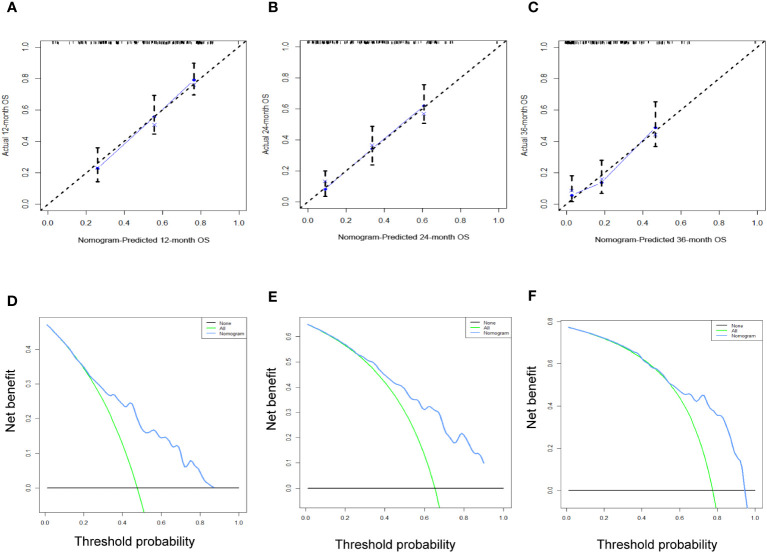
The calibration curves **(A–C)** and the DCA curves **(D–F)** of the prognostic nomogram for the 1-year, 2-year, and 3-year in the external validation set.

**Table 3** displays the baseline characteristics of the aged individuals with ECRLM. There exists significant difference in race and chemotherapy between the two groups, potentially because of variations in geographical differences and insufficient sample size. The prognostic factors for OS of patients with ECRLM were identified using univariate and multivariate Cox analyses, which identified 10 independent prognostic factors, including age at diagnosis, CEA level, tumor size, lung metastasis, bone metastasis, chemotherapy, surgery, N stage, grade, and race (**Table 4**). A prediction model was produced using these independent prognostic variables to determine the OS at 12, 24, and 36 months in patients with ECRLM (**Figure 6**). In addition, the prognostic nomogram was subjected to internal and external validation. In internal validation, the AUC of the prognostic nomogram for 1-, 2-, and 3-year OS were 0.787, 0.777, and 0.765, respectively, in the training cohort and 0.789, 0.739, and 0.732, respectively, in the validation cohort, according to the ROC curves (**Figures 7A, B**). The calibration curves for the 1-year, 2-year, and 3-year cohorts demonstrated a high degree of concordance between the forecast and real survival in both cohorts (**Figures 8A–F**). According to the DCA results, the nomogram was clinically useful in predicting 1-, 2-, and 3-year OS in both cohorts (**Figures 9A–F**). Additionally, computations were conducted to ascertain the cumulative points for all patients using a prognostic nomogram. Subsequently, we used X-tile software to determine two-threshold points for all patient scores, which were used to separate the recipients into three distinct groups for Kaplan–Meier survival analysis. As illustrated in **Figure 10**, patients in the high-risk group experienced considerably inferior survival outcomes compared to those in the median- and low-risk groups. During external validation, the nomogram achieved AUC of 0.703, 0.708, and 0.684 for 12-, 24-, and 36-month OS, respectively (**Figure 11**). The calibration curves for the 12-, 24-, and 36-month exhibited a strong level of agreement between the predicted and actual survival rates (**Figures 12A–C**). DCA demonstrated that this nomogram has the potential as a valuable clinical measure for prognosticating OS in patients with ECRLM (**Figures 12D–F**).

**Table 3:** The baseline data of ECRLM patients.

Characteristics	Number of patients (n, %)				
	Total N=3012	Training cohort N=2018	Inclusion Validation N=994	External Validation N=188	p
Age					
65-75	1700(56.44)	1162(57.58)	538(54.12)	104 (55.32)	0.763
>75	1312 (43.54)	856(42.42)	456(45.88)	84(44.68)	
Insurance status					
Insured	2989(99.24)	2005(99.36)	984(98.99)	186(98.94)	0.65
Uninsured	23(0.76)	13(0.64)	10(1.01)	2(1.06)	
Marital status					
Married	1660 (55.11)	1113(55.15)	547(55.03)	107(56.91)	0.63
Unmarried ^a^	1352(44.89)	905(44.85)	447(44.97)	81(43.09)	
Gender					
Female	1414(46.95)	941(46.63)	473(47.59)	82(43.62)	0.375
Male	1598(53.05)	1077(53.37)	521(52.41)	106(56.38)	
Race					
Black	391(12.98)	310(15.36)	81(8.15)	0(0)	0
Other ^b^	278(9.23)	164(8.13)	114(11.47)	188(100)	
White	2343(77.79)	1544(76.51)	799(80.38)	0(0)	
Primary site					
Left	1052(34.93)	711(35.23)	341(34.31)	71(37.77)	0.671
Rectum	331(10.99)	220(10.90)	111(11.17)	18(9.57)	
Right	1629(54.08)	1087(53.87)	542(54.53)	99(52.66)	
Grade					
I	137(4.55)	90(4.46)	47(4.73)	4(2.13)	0.174
II	2031(67.43)	1375(68.14)	656(66.00)	128(68.09)	
III	700(23.24)	447(22.15)	253(25.45)	42(22.34)	
IV	144(4.78)	106(5.25)	38(3.82)	14(7.45)	
Histology					
Adenocarcinoma	2452(81.41)	1640(81.27)	812(81.69)	150(79.79)	0.58
Other	560(18.59)	378(18.73)	182(18.31)	38(20.21)	
T stage					
T1	198(6.57)	143(7.09)	55(5.53)	19(10.11)	0.129
T2	85(2.82)	60(2.97)	25(2.52)	2(1.06)	
T3	1637(54.35)	1075(53.27)	562(56.54)	104(55.32)	
T4	1092(36.25)	740(36.67)	352(35.41)	63(33.51)	
N stage					
N0	694(23.04)	478(23.69)	216(21.73)	42(22.34)	0.852
N1	1131(37.55)	782(38.75)	349(35.11)	68(36.17)	
N2	1187(39.41)	758(37.56)	429(43.16)	78(41.49)	
Surgery					
No	437(14.51)	303(15.01)	134(13.48)	33(17.55)	0.253
Yes	2575(85.49)	1715(84.99)	860(86.52)	155(82.45)	
Chemotherapy					
No/Unknown	1164(38.65)	743(36.82)	421(42.35)	98(52.13)	0
Yes	1848(61.35)	1275(63.18)	573(57.65)	90(47.87)	
Tumor size					
≤5cm	1572(63.12)	1059(52.48)	513(51.61)	103(54.79)	0.489
>5cm	1440(36.88)	959(47.52)	481(48.39)	85(45.21)	
Bone metastasis					
No	2927(99.69)	1964(97.32)	963(96.88)	182(96.81)	0.767
Yes	85(0.31)	54(2.68)	31(3.12)	6(3.19)	
Brain metastasis					
No	2995(99.91)	2005(99.36)	990(99.60)	187(99.47)	1
Yes	17(0.09)	13(0.64)	4(0.40)	1(0.53)	
Lung metastasis					
No	2487(98.10)	1666(82.56)	821(82.60)	152(80.85)	0.548
Yes	525(1.90)	352(17.44)	173(17.40)	36(19.15)	
CEA					
Negative	498(16.53)	334(16.55)	164(16.50)	38(20.21)	0.19
positive	2514(83.47)	1684(83.45)	830(83.50)	150(79.79)	

a Includes single, separated, widowed, and divorced.

b Includes American Indian/Alaska Native and Asian or Pacific Islander.

**Table 4:** Univariate and multivariate Cox regression analysis for identification independent prognostic factors in ECRLM patients.

Variables	Univariate analysis			Multivariate analysis		
	HR	95%CIs	P	HR	95%CIs	P
Age						
65-75	Reference			Reference		
>75	1.73	1.57-1.92	0	1.37	1.23-1.53	0
Bone metastasis						
NO	Reference			Reference		
Yes	1.96	1.48-2.61	0	1.7	1.26-2.28	<0.001
Brain metastasis						
NO	Reference			Reference		
Yes	2.32	1.34-4.01	0.003	1.17	0.67-2.05	0.589
CEA						
Negative	Reference			Reference		
Positive	1.57	1.36-1.82	0	1.67	1.44-1.94	0
Chemotherapy						
No	Reference			Reference		
Yes	0.31	0.28-0.34	0	0.32	0.29-0.36	0
Grade						
I	Reference			Reference		
II	1.15	0.89-1.49	0.281	1.03	0.79-1.33	0.8398
III	1.88	1.43-2.45	0	1.48	1.13-1.95	0.0048
IV	2.4	1.74-3.31	0	1.79	1.29-2.49	<0.01
Histology						
Adenocarcinoma	Reference			Reference		
Other	1.09	0.95-1.23	0.212	NA		NA
Insurance						
Insured	Reference			Reference		
Uninsured	0.86	0.41-1.81	0.694	NA		NA
Lung metastasis						
No	Reference			Reference		
Yes	1.4	1.23-1.59	0	1.22	1.07-1.39	0.0037
Marital status						
Married	Reference			Reference		
Unmarried ^a^	1.32	1.2-1.46	0	1.06	0.95-1.19	0.2713
N stage						
N0	Reference			Reference		
N1	1.06	0.93-1.22	0.389	1.28	1.1-1.48	<0.05
N2	1.39	1.22-1.59	0	1.64	1.41- 1.9	0
Primary site						
Left	Reference			Reference		
Rectum	0.92	0.76-1.1	0.352	0.86	0.7- 1.05	0.1418
Right	1.29	1.16-1.44	0	1.1	0.98-1.23	0.1046
Race						
Black	Reference			Reference		
Other ^b^	0.81	0.65-1.01	0.065	0.72	0.58-0.91	0.0051
White	0.87	0.76-0.99	0.04	0.91	0.79-1.04	0.167
Sex						
Female	Reference			Reference		
Male	0.83	0.75-0.91	0	1.02	0.91-1.13	0.7641
Surgery						
No	Reference			Reference		
Yes	0.66	0.58-0.76	0	0.47	0.38-0.57	0
T stage						
T1	Reference			Reference		
T2	0.39	0.26-0.59	0	0.6	0.39-0.91	0.17
T3	0.68	0.56-0.83	0	0.91	0.71-1.16	0.4317
T4	1.04	0.85-1.28	0.677	1.23	0.96-1.58	0.1079
Tumor size						
≤5cm	Reference			Reference		
>5cm	1.26	1.14-1.39	0	1.13	1.02-1.26	0.0183

a Includes single, separated, widowed, and divorced.

b Includes American Indian/Alaska Native and Asian or Pacific Islander.

## 4 Discussion

Age-related diseases, encompassing a wide array of medical conditions such as diabetes, respiratory failure, and heart failure, have become common among the senior demographic. These diseases can have a substantial impact on the prevention, detection, and treatment of malignant tumors. In contrast to non-aged patients, patients with ECRC have a distinct set of characteristics that encompass not only TNM staging but also economic condition, marital status, and cognitive level of cancer, all of which influence the probability and prognosis of ECRLM. Therefore, it may be impractical to strictly adhere to diagnostic and therapeutic approaches adapted for the overall population when dealing with older individuals with intricate comorbidities or cognitive deficits. Regrettably, few studies have considered patients with ECRC as a distinct cohort to assess their risk of liver metastasis and long-term prognosis. Conversely, the majority of research has focused on either all patients, young patients, or metastases in the lungs, bones, and brain (18–22). Our study, which focused on patients with ECRC and explored the prevalence and prognosis of LM in this population, is a pioneering effort in the field of CRC research. Following analysis of the independent risk and prognostic variables related to patients with ECRLM, two nomograms were established that could successfully predict the occurrence and prognosis of synchronous LM.

This study showed that age at diagnosis, marital status, race, sex, bone metastases, lung metastases, CEA level, tumor size, Grade, histology, primary site, T stage, and N stage were strongly linked to the occurrence of LM.

In 1990, Bufill et al. demonstrated for the first time, from a molecular genetic perspective, that the left and right colorectal halves differed significantly in embryonic development, immunology, pathology, microenvironment, and blood supply (23). According to our research, colon cancer is more likely to metastasize to the liver than rectal cancer. Zhu et al. offered a rationale for this observation, highlighting that intestinal mesenteric drainage from colon tumors typically flows into the portal vein of the liver. Consequently, colon cancer frequently spreads to the liver. However, since the venous blood from the rectum enters the systemic circulation, it is common for rectal tumors to metastasize to the lungs (24). LM was more prevalent in the left colon than in the right colon, which is consistent with the results of previous studies (25, 26). However, the relationship between primary tumor site and liver metastasis requires further investigation.

According to our results, marital status was an independent risk factor for ECRLM. The older patients who have lost the support of their families or loved ones are more likely to experience anxiety. Cancer accompanied by anxiety or depression can aggravate treatment-related side effects, diminish treatment efficacy, and promote tumor recurrence and metastasis (27). However, additional investigations are necessary to substantiate this assertion in future studies.

In high-risk populations, particularly those with undefined low-density liver lesions, Positron Emission Tomography (PET-CT) and invasive procedures such as needle biopsy are also required to make a definitive diagnosis. These examinations are costly and, as routine screening procedures, they can place a substantial financial burden on patients. Our nomogram can quickly screen high-risk populations for CRCLM, and targeted examinations for these patients can not only avoid delaying the timing of radiofrequency ablation and surgery but also lessen the economic burden on patients.

Prognostic factors are of great significance for guiding individualized treatment and improving survival rates. This study proved that the OS of ECRCLM patients can be affected by 10 variables, such as age at diagnosis, CEA level, and lung metastases, ect.

Our study found that the degree of tumor differentiation is a crucial factor in determining the survival of these patients. The lower the degree of differentiation, the higher the malignancy and the poorer the clinical treatment efficacy, resulting in a poorer prognosis. Due to their high invasiveness and motility, poorly differentiated cancer cells are prone to shedding into the circulation and infiltrating the liver, thereby metastasizing. Moreover, poorly differentiated or undifferentiated cancer cells are more likely to generate microscopic tumor thrombi that rapidly disseminate throughout the liver and negatively affect long-term survival. Further case studies are required to demonstrate whether the degree of cell differentiation in primary malignant tumors has an independent effect on the prognosis of patients with ECRLM, as there are currently conflicting research results.

There is a significant correlation between lymph node metastasis and prognosis, and patients with more lymph node metastases have an inferior postoperative prognosis, as shown in previous research (28).

In our study, multivariate COX analysis revealed that CEA was a significant prognostic factor influencing patient prognosis, which is consistent with earlier findings (29, 30). We attempted to describe this phenomenon as follows: (1) CEA can bind to liver cells and function as a receptor for adhesion to circulating cancer cells, owing to its adhesion activity. (2) Excessive CEA secretion can result in unstable connections between cancer cells, disordered arrangement, and loss of polarity, which promote the migration and discharge of cancer cells and their entry into circulation. (3) CEA is an endogenous immunosuppressive agent that can inhibit host- and non-specific immune responses and help cancer cells evade immune surveillance. The role of CEA in the pathogenesis of LM may be complex and additional research is required.

The most effective treatment for CRC patients with LM (CRLM) is radical resection of the primary tumor and liver metastases (31). Even in situations in which liver metastases cannot be removed, eradication of the primary lesion has survival benefits (32). In comparison to younger individuals, the physical condition of those over 65 years of age is weaker, and whether they can tolerate surgery remains controversial. A recent meta-analysis involving 14 retrospective studies on patients with ECRLM undergoing surgery between 1994 and 2016 revealed that the postoperative mortality rates for patients aged > 70 years were 4% and for patients over 75 years of age was 6% (33). These outcomes were considered acceptable.

Unfortunately, only 10–20% of patients diagnosed with CRLM can undergo radical surgery (34). Patients with unresectable CRLM are primarily treated with systemic chemotherapy to increase their overall survival (35). Preoperative neoadjuvant therapy can reduce the size, quantity, and distribution of primary and metastatic tumors, thereby improving resectability, whereas postoperative adjuvant therapy can reduce the recurrence rate and improve prognosis (36, 37). Our study demonstrated that chemotherapy can affect patient prognosis.

In a retrospective study involving 13,662 patients with liver metastasis from CRC, Bai et al. found that the prognosis of CRLM with extrahepatic metastasis was poor (38). The lungs are commonly identified as the primary location of extrahepatic distant metastases in patients with CRC (39). In many cases, older patients often experience respiratory diseases. In the presence of lung metastases, further diminished lung function renders patients unable to tolerate surgery and chemotherapy, thereby reducing the chances of treatment. In patients with CRLM, bone metastasis is regarded as a disease progression marker, indicating that the tumor has a more aggressive and malignant biological behavior. Problems associated with bone metastasis include severe discomfort, pathologically broken bones, hypercalcemia, and nerve squeezing, all of which significantly shorten a patient’s lifespan. The present study revealed that the presence of lung and bone metastases had a detrimental effect on the survival outcomes of patients with ECRLM.

Accompanying a greater awareness of the genetic drivers of tumor biology, there is speculation regarding the potential correlation between certain molecular cancer biomarkers, such as ras and braf, and the incidence and prognostic implications of CRLM (40). The occurrence of KRAS mutations among CRC exceeds 50%, whereas the occurrence of HRAS and NRAS mutations is rather infrequent (41). In a study of Chinese patients with CRLM, the KRAS gene exhibits a notably high incidence of mutations, meanwhile, the RAS gene serves as an independent factor that influences the prognosis of CRLM (42).However, Roya et al. did a study whereby they performed Kaplan Meier survival estimates on a sample of 173 patients with CRLM. The findings of their analysis indicated that there was no statistically significant disparity in OS between patients with KRAS mutant genes and those with wild-type genes (43). There is ongoing debate over the influence of RAS on the prognosis of CRLM. Additional investigation is required to gain a more comprehensive understanding of this topic.

Research conducted in China has revealed contrasting clinical characteristics between Chinese and Western patients with BRAF mutations in metastatic CRC. These disparities mostly emerge as an earlier beginning age and a reduced occurrence of microsatellite instability among Chinese patients (40). In a multicenter retrospective study conducted in China, it was observed that BRAF V600E, the most common kind of BRAF mutation, is the key factor impacting OS in patients with CRLM (44).

Moreover, a number of biomarkers, including human epidermal growth factor receptor 2 (HER2) amplification, as well as microsatellite instability (MSI) or mismatch repair (MMR) play important roles in the occurrence and development of CRC (45). Further research about these biomarkers is needed to aid clinicians in determining the most appropriate course of treatment.

This study has multiple benefits compared with previous studies. Notably, our data underwent both external and internal validation, thereby enhancing the reliability of the nomograms. Furthermore, the utilization of nomograms can effectively mitigate doctor-patient disagreements arising from the ambiguous prognostic information of patients with ECRLM. It cannot be overstated that these nomograms can serve as a valuable tool for facilitating follow-up procedures, accordingly strengthening the management of long-term treatment for patients with ECRLM.

While acknowledging the various merits of this study, it is also imperative to consider its limitations. The SEER database has some limitations that prevent the inclusion of critical clinical variables, such as blood test results, information on targeted treatment, and gene expression details, all of which have the potential to influence the development and outcome of LM. Meanwhile, it is essential to weigh the conceivable repercussions of selection bias on the results of this study given its retrospective methodology. In addition, although external validation of the nomogram can aid in reducing model overfitting, the case resources and sample size of our external validation cohort may have been inadequate. Large samples from multicenter cohorts worldwide are required to enhance the external validation.

## 5 Conclusion

In our study, two easy-to-use nomograms may help surgeons devise a more efficient, individualized treatment plan for ECRC patients at a high risk of LM by identifying such patients and estimating their survival.

## Data availability statement

Publicly available datasets were analyzed in this study. This data can be found here: https://seer.cancer.gov/seerstat.

## Ethics statement

The studies involving humans were approved by Ethics committee of China-Japan Union Hospital of Jilin University. The studies were conducted in accordance with the local legislation and institutional requirements. This study is retrospective and anonymous, therefore obtaining the patient’s written informed permission in a timely manner is difficult.

## Author contributions

QW: Data curation, Investigation, Validation, Writing – original draft. KS: Data curation, Investigation, Methodology, Writing – review & editing. BF: Data curation, Investigation, Methodology, Writing – review & editing. MW: Data curation, Writing – review & editing. ZX: Supervision, Validation, Writing – review & editing.

## References

[1] Sung H Ferlay J Siegel RL Laversanne M Soerjomataram I Jemal A. Global cancer statistics 2020: GLOBOCAN estimates of incidence and mortality worldwide for 36 cancers in 185 countries. CA Cancer J Clin (2021) 71(3):209–49. doi: 10.3322/caac.21660 33538338

[2] Smetana K Jr. Lacina L Szabo P Dvorankova B Broz P Sedo A. Ageing as an important risk factor for cancer. Anticancer Res (2016) 36(10):5009–17. doi: 10.21873/anticanres.11069 27798859

[3] Kuipers EJ Grady WM Lieberman D Seufferlein T Sung JJ Boelens PG. Colorectal cancer. Nat Rev Dis Primers. (2015) 1:15065. doi: 10.1038/nrdp.2015.65 27189416 PMC4874655

[4] Kim JH. Chemotherapy for colorectal cancer in the elderly. World J Gastroenterol (2015) 21(17):5158–66. doi: 10.3748/wjg.v21.i17.5158 PMC4419056 25954089

[5] Quaglia A Tavilla A Shack L Brenner H Janssen-Heijnen M Allemani C. The cancer survival gap between elderly and middle-aged patients in Europe is widening. Eur J Cancer (Oxford Engl 1990). (2009) 45(6):1006–16. doi: 10.1016/j.ejca.2008.11.028 19121578

[6] Luo T Wang Y Shan X Bai Y Huang C Li G. Nomogram based on homogeneous and heterogeneous associated factors for predicting distant metastases in patients with colorectal cancer. World J Surg Oncol (2021) 19(1):30. doi: 10.1186/s12957-021-02140-6 33504354 PMC7842036

[7] Rawla P Sunkara T Barsouk A. Epidemiology of colorectal cancer: incidence, mortality, survival, and risk factors. Prz Gastroenterol (2019) 14(2):89–103. doi: 10.5114/pg.2018.81072 31616522 PMC6791134

[8] Vallance AE Young AL Kuryba A Braun M Hill J Jayne DG. The impact of advancing age on incidence of hepatectomy and post-operative outcomes in patients with colorectal cancer liver metastases: a population-based cohort study. HPB Off J Int Hepato Pancreato Biliary Assoc (2019) 21(2):167–74. doi: 10.1016/j.hpb.2018.06.1808 30076012

[9] Stewart CL Warner S Ito K Raoof M Wu GX Kessler J. Cytoreduction for colorectal metastases: liver, lung, peritoneum, lymph nodes, bone, brain. When does it palliate, prolong survival, and potentially cure? Curr problems surgery. (2018) 55(9):330–79. doi: 10.1067/j.cpsurg.2018.08.004 PMC6422355 30526930

[10] van der Geest LG Lam-Boer J Koopman M Verhoef C Elferink MA de Wilt JH. Nationwide trends in incidence, treatment and survival of colorectal cancer patients with synchronous metastases. Clin Exp metastasis. (2015) 32(5):457–65. doi: 10.1007/s10585-015-9719-0 25899064

[11] Snyder RA Hao S Irish W Zervos EE Tuttle-Newhall JE Parikh AA. Thirty-Day Morbidity after simultaneous resection of colorectal cancer and colorectal liver metastasis: American College of Surgeons NSQIP analysis. J Am Coll Surg (2020) 230(4):617–27.e9. doi: 10.1016/j.jamcollsurg.2019.12.018 32007534

[12] Marín Hernández C Robles Campos R Pérez Flores D López Conesa A Parrilla Paricio P. Prognostic factors after resection of colorectal cancer liver metastases. Cirugia espanola. (2009) 85(1):32–9. doi: 10.1016/s0009-739x(09)70084-3 19239935

[13] Gong B Kao Y Zhang C Sun F Gong Z Chen J. Identification of hub genes related to carcinogenesis and prognosis in colorectal cancer based on integrated bioinformatics. Mediators Inflamm (2020) 2020:5934821. doi: 10.1155/2020/5934821 32351322 PMC7171686

[14] Balachandran VP Gonen M Smith JJ DeMatteo RP. Nomograms in oncology: More than meets the eye. Lancet Oncol (2015) 16(4):e173–80. doi: 10.1016/S1470-2045(14)71116-7 PMC4465353 25846097

[15] Adam R de Gramont A Figueras J Kokudo N Kunstlinger F Loyer E. Managing synchronous liver metastases from colorectal cancer: a multidisciplinary international consensus. Cancer Treat Rev (2015) 41(9):729–41. doi: 10.1016/j.ctrv.2015.06.006 26417845

[16] Pak MG Koh HJ Roh MS. Clinicopathologic significance of TRAP1 expression in colorectal cancer: A large scale study of human colorectal adenocarcinoma tissues. Diagn Pathol (2017) 12(1):6. doi: 10.1186/s13000-017-0598-3 28088229 PMC5237536

[17] Liang Y Li Q He D Chen Y Li J. Tumor size improves the accuracy of the prognostic prediction of T4a stage colon cancer. Sci Rep (2021) 11(1):16264. doi: 10.1038/s41598-021-95828-4 34381141 PMC8357783

[18] Cheng X Li Y Chen D Xu X Liu F Zhao F. Nomogram predicting the survival of young-onset patients with colorectal cancer liver metastases. Diagnostics (Basel) (2022) 12(6):1395. doi: 10.3390/diagnostics12061395 35741205 PMC9221975

[19] Tang M Wang H Cao Y Zeng Z Shan X Wang L. Nomogram for predicting occurrence and prognosis of liver metastasis in colorectal cancer: A population-based study. Int J Colorectal Dis (2021) 36(2):271–82. doi: 10.1007/s00384-020-03722-8 32965529

[20] Deng S Jiang Z Cao Y Gu J Mao F Xue Y. Development and validation of a prognostic scoring system for patients with colorectal cancer hepato-pulmonary metastasis: A retrospective study. BMC Cancer. (2022) 22(1):643. doi: 10.1186/s12885-022-09738-3 35690752 PMC9188712

[21] Zhenghong Zihua Z Guoweijian Zhangning Caiyunyun Yingjiangshan. Retrospective study of predictors of bone metastasis in colorectal cancer patients. J Bone Oncol (2017) 9:25–8. doi: 10.1016/j.jbo.2017.10.003 PMC5715439 29234589

[22] Li W Wang T Zhu Y Yu H Ma L Ding Y. Brain metastasis from colorectal cancer: Treatment, survival, and prognosis. Medicine. (2022) 101(40):e30273. doi: 10.1097/md.0000000000030273 36221357 PMC9542566

[23] Bufill JA. Colorectal cancer: evidence for distinct genetic categories based on proximal or distal tumor location. Ann Internal Med (1990) 113(10):779–88. doi: 10.7326/0003-4819-113-10-779 2240880

[24] Zhu YJ Chen Y Hu HY Zhou YW Zhu YT Liu JY. Predictive risk factors and online nomograms for synchronous colon cancer with liver metastasis. Front Oncol (2020) 10:1681. doi: 10.3389/fonc.2020.01681 33123459 PMC7566411

[25] Norén A Eriksson HG Olsson LI. Selection for surgery and survival of synchronous colorectal liver metastases; a nationwide study. Eur J Cancer (Oxford Engl 1990) (2016) 53:105–14. doi: 10.1016/j.ejca.2015.10.055 26702764

[26] Engstrand J Nilsson H Strömberg C Jonas E Freedman J. Colorectal cancer liver metastases - a population-based study on incidence, management and survival. BMC Cancer. (2018) 18(1):78. doi: 10.1186/s12885-017-3925-x 29334918 PMC5769309

[27] Lee MJ Huang CW Lee CP Kuo TY Fang YH Chin-Hung Chen V. Investigation of anxiety and depressive disorders and psychiatric medication use before and after cancer diagnosis. Psycho-oncology. (2021) 30(6):919–27. doi: 10.1002/pon.5672 33724591

[28] Han S Wu W Da M Xu J Zhuang J Zhang L. Adequate lymph node assessments and investigation of gut microorganisms and microbial metabolites in colorectal cancer. Onco Targets Ther (2020) 13:1893–906. doi: 10.2147/ott.S242017 PMC7061441 32184624

[29] Arru M Aldrighetti L Castoldi R Di Palo S Orsenigo E Stella M. Analysis of prognostic factors influencing long-term survival after hepatic resection for metastatic colorectal cancer. World J surgery. (2008) 32(1):93–103. doi: 10.1007/s00268-007-9285-y 18027020

[30] Cady B Jenkins RL Steele GD Jr. Lewis WD Stone MD McDermott WV. Surgical margin in hepatic resection for colorectal metastasis: A critical and improvable determinant of outcome. Ann Surg (1998) 227(4):566–71. doi: 10.1097/00000658-199804000-00019 PMC1191314 9563547

[31] Balachandran VP Arora A Gönen M Ito H Turcotte S Shia J. A validated prognostic multigene expression assay for overall survival in resected colorectal cancer liver metastases. Clin Cancer Res an Off J Am Assoc Cancer Res (2016) 22(10):2575–82. doi: 10.1158/1078-0432.Ccr-15-1071 PMC4978939 26733613

[32] Faron M Pignon JP Malka D Bourredjem A Douillard JY Adenis A. Is primary tumour resection associated with survival improvement in patients with colorectal cancer and unresectable synchronous metastases? A pooled analysis of individual data from four randomised trials. Eur J Cancer (Oxford Engl 1990). (2015) 51(2):166–76. doi: 10.1016/j.ejca.2014.10.023 25465185

[33] van Tuil T Dhaif AA Te Riele WW van Ramshorst B van Santvoort HC. Systematic review and meta-analysis of liver resection for colorectal metastases in elderly patients. Digestive surgery. (2019) 36(2):111–23. doi: 10.1159/000487274 29502126

[34] Isoniemi H Osterlund P. Surgery combined with oncological treatments in liver metastases from colorectal cancer. Scandinavian J Surg SJS Off Organ Finnish Surg Soc Scandinavian Surg Society. (2011) 100(1):35–41. doi: 10.1177/145749691110000107 21482503

[35] Benson AB 3rd Venook AP Cederquist L Chan E Chen YJ Cooper HS. Colon cancer, version 1.2017, NCCN clinical practice guidelines in oncology. J Natl Compr Cancer Network JNCCN (2017) 15(3):370–98. doi: 10.6004/jnccn.2017.0036 28275037

[36] Chow FC Chok KS. Colorectal liver metastases: An update on multidisciplinary approach. World J hepatology. (2019) 11(2):150–72. doi: 10.4254/wjh.v11.i2.150 PMC6393711 30820266

[37] Saad AM Abdel-Rahman O. Initial systemic chemotherapeutic and targeted therapy strategies for the treatment of colorectal cancer patients with liver metastases. Expert Opin pharmacotherapy. (2019) 20(14):1767–75. doi: 10.1080/14656566.2019.1642324 31314604

[38] Bai S Chen L Zhu G Xuan W Hu F Liu W. Prognostic value of extrahepatic metastasis on colon cancer with liver metastasis: A retrospective cohort study. Front Oncol (2023) 13:1172670. doi: 10.3389/fonc.2023.1172670 37346071 PMC10280983

[39] Cao H Xu E Liu H Wan L Lai M. Epithelial-mesenchymal transition in colorectal cancer metastasis: A system review. Pathology Res practice. (2015) 211(8):557–69. doi: 10.1016/j.prp.2015.05.010 26092594

[40] Xu T Li J Wang Z Zhang X Zhou J Lu Z. Real-world treatment and outcomes of patients with metastatic BRAF mutant colorectal cancer. Cancer Med (2023) 12(9):10473–84. doi: 10.1002/cam4.5783 PMC10225206 36912150

[41] Kitsel Y Cooke T Sotirchos V Sofocleous CT. Colorectal cancer liver metastases: Genomics and biomarkers with focus on local therapies. Cancers (2023) 15(6):1679. doi: 10.3390/cancers15061679 36980565 PMC10046329

[42] Li Z Chen Y Wang D Wang G He L Suo J. Detection of KRAS mutations and their associations with clinicopathological features and survival in Chinese colorectal cancer patients. J Int Med Res (2012) 40(4):1589–98. doi: 10.1177/147323001204000439 22971512

[43] Dolatkhah R Dastgiri S Eftekhar Sadat AT Farassati F Nezamdoust M Somi MH. Impact of RAS/RAF mutations on clinical and prognostic outcomes in metastatic colorectal cancer. BioImpacts BI. (2021) 11(1):5–14. doi: 10.34172/bi.2021.02 33469503 PMC7803924

[44] Han L Xue J Wu X Liang G Wu Y Guo F. Multidisciplinary approach to the diagnosis and treatment of patients with potentially resectable colorectal cancer liver metastasis: Results of a multicenter study. Ann palliative Med (2022) 11(2):717–29. doi: 10.21037/apm-22-87 35249349

[45] Martelli V Pastorino A Sobrero AF. Prognostic and predictive molecular biomarkers in advanced colorectal cancer. Pharmacol Ther (2022) 236:108239. doi: 10.1016/j.pharmthera.2022.108239 35780916

